# Canine caliciviruses of four serotypes from military and research dogs recovered in 1963−1978 belong to two phylogenetic clades in the *Vesivirus* genus

**DOI:** 10.1186/s12985-018-0944-4

**Published:** 2018-02-23

**Authors:** Leonard N. Binn, Erica A. Norby, Ruth H. Marchwicki, Richard G. Jarman, Paul B. Keiser, Jun Hang

**Affiliations:** Viral Diseases Branch, Walter Reed Army Institute of Research, Silver Spring, MD 20910 USA

**Keywords:** Calicivirus, Vesivirus, Canine calicivirus, Viral infection, Military dog, Animal virus

## Abstract

**Background:**

Vesiviruses (family *Caliciviridae*) had been shown capable of invading a variety of host species, raising concern of their zoonotic potential. Since the 1980’s, several canine caliciviruses (CaCV) isolates have been reported and are phylogenetically related to the vesiviruses with features distinct from both *Vesicular exanthema of swine virus* (VESV) and *Feline calicivirus* (FCV) species in phylogeny, serology and cell culture specificities. Etiological studies of canine diseases in dogs used for military services and laboratory studies were conducted in 1963–1978 at the Walter Reed Army Institute of Research. Multiple known and unknown viral pathogens including caliciviruses were recovered.

**Methods:**

Four unidentified isolates were recovered in Walter Reed Canine Cells (WRCC) from respiratory, fecal and penile specimens. Physicochemical tests, electron microscopy, viral cultivation in human and animal cells, antibody neutralization assays, and recently the genome sequencing were used to characterize the isolates. Sera from these dogs and their cohorts were tested with the isolates to determine origin and prevalence of the infections.

**Results:**

The viral isolates were small non-enveloped spherical RNA virions, 27 to 42 nm in diameter with cup-like structures, indicating they are caliciviruses. They propagated in WRCC and MDCK cells, not in either other canine cells or human and other animal cells. Each isolate is antigenically distinct and react with dog sera in respective cohorts. The genomes have nucleotide identities ranging from 70.3% to 90.7% and encode the non-structural polyprotein (1810 amino acids), major capsid protein (691 amino acids) and minor structural protein (134 amino acids). They belong to two different phylogenetic clades in *Vesivirus* genus with close relation with canine calicivirus (CaCV).

**Conclusions:**

These CaCV isolates have restricted cell tropism, antigenic diversity and genetic variation. Further investigation will shed light on antigenic relation to other vesiviruses, and its pathogenicity for dogs and potential infectivity to other animals. Together with the previously reported CaCV strains provides significant evidence to support the formation of a new CaCV species in the *Vesivirus* genus.

**Electronic supplementary material:**

The online version of this article (10.1186/s12985-018-0944-4) contains supplementary material, which is available to authorized users.

## Background

The caliciviruses (family *Caliciviridae*) are non-enveloped, positive sense, single-stranded RNA viruses with diameters ranging from 27 to 40 nm. Caliciviruses cause a wide range of significant diseases in human and animals. At present, there are five recognized genera, i.e., *Norovirus*, *Sapovirus*, *Lagovirus*, *Vesivirus*, and *Nebovirus* with several additional candidate genera or species proposed and under evaluation by the International Committee on Taxonomy of Viruses (ICTV) [1, 2] (http://www.caliciviridae.com/unclassified/unclassified.htm). In the *Vesivirus* genus, *Vesicular exanthema of swine virus* (VESV) and *Feline calicivirus* (FCV) are two species currently approved by ICTV. Several canine caliciviruses (CaCV) isolates have been identified and shown to be phylogenetically related to vesiviruses with features distinct from both VESV and FCV in phylogeny, serology and cell culture specificities. CaCV is a probable species in the *Vesivirus* genus, as stated by ICTV [2]. It is still unclassified to date and the evidence presented herein should facilitate the classification and acceptance of CaCV as a species of vesivirus.

Many viruses found in human and other animal species can also infect dogs asymptomatically or cause respiratory, digestive, neurologic and genital diseases with mild to severe symptoms. In response to the use of dogs in military services and laboratory studies, etiological studies of canine diseases were conducted in 1963–1978 at the Walter Reed Army Institute of Research (WRAIR) [3, 4]. In addition to several known canine viral pathogens [5, 6], four unidentified viruses were recovered in Walter Reed Canine Cells (WRCC) producing similar cytopathic effects (CPE). The isolates were not recognized by available human and dog reference virus antisera. Studies of their physicochemical properties and electron microscope observations identified the isolates as likely caliciviruses. Our recent whole genome sequencing of these canine isolates clearly identified them as vesiviruses and elucidated their genetic relationships to the other members of the *Caliciviridae* family. We herein report the viral isolation and characterization results, which were made in 1963–1978 canine diseases etiological study but were not published, and additional genomics analysis supporting the serological diversity of CaCV strongly suggesting that these isolates and similar CaCV are a unique species within *Vesivirus* genus [7–9].

## Methods

Collection of the specimens, viral isolations, physicochemical characterization of the viruses and the serological assays were performed in the period of 1963–1978. The purification and genome sequencing of the nucleic acids from the archived viral cultures were done recently.

### Specimens

Dog throat, rectal and penile swabs were collected, placed in 2–5 ml of veal infusion broth transport media (Difco Laboratories Inc., Detroit, MI) and frozen at < -60 °C until processed for virus isolation and identification [4]. Blood specimens were collected from each dog and dogs in each cohort at the time and 14–28 days later.

### Cell culture and isolation of viruses

The WRCC [6] and primary dog kidney cells (PDK) were prepared in our laboratory; other primary and continuous cells were obtained from commercial sources (Microbiological Associates, Bethesda, Maryland, currently, Lonza, Walkersville, MD). The WRCC were cultured at 35 °C with Medium 199 containing 10% fetal bovine serum, 100 units/ml of penicillin, 100 μg/ml of streptomycin and 2.5 μg/ml of amphotericin B. Cultures for virus studies were maintained in Basal Media Eagle with 2% fetal bovine serum, 1% L-glutamine, and 100 units/ml of penicillin, 100 μg/ml of streptomycin, and 2.5 μg/ml of amphotericin B. Specimens producing cytopathic effects (CPE) were chosen for further study after purification by three terminal dilutions in WRCC. Seed virus preparations were made for virus characterization testing and identification. Virus titrations were done in WRCC.

### Determination of isolates’ physicochemical properties

The procedures to determine the presence of a viral envelope by chloroform/ether treatment, type of nucleic acid employing 5-iodo-2-deoxyuridine (IUDR), acid stability (pH 3.0) and heat stability (MgCl_2_) and size employing membranes of graded porosity are described elsewhere [10].

Prior to negative staining, the virus was partially purified by differential centrifugation at low speed of approximate 10,000×g and high speeds of approximate 100,000×g. The purified virions were stained with 2% phosphotungstic acid and were examined with a Hitachi HU 12 electron microscope. Buoyant density was determined by adding the concentrated virus either by layering or mixing with cesium chloride solution with a refractive index of 1.380 and centrifugation at 33,000 rpm in the SW39 head for 20 h in a Beckman model L ultracentrifuge. After ultracentrifugation, fractions were collected for infectivity and density determinations. For control purposes poliovirus type 1 was tested at the same time and had the expected value of 1.33 g/ml.

### Neutralization tests

The neutralization tests were done in WRCC as previously described [4, 11]. Reference antisera for the viral isolates were prepared in rabbits given multiple doses of virus prepared from infected cells maintained in serum-free media. All the pre-immunization sera were free of neutralizing activity to the immunizing virus. Picornavirus and reovirus reference antisera were obtained from the NIH Reference Reagent Program, National Institute of Allergy and Infectious Diseases, Bethesda MD. In addition, antisera to dog viruses were obtained from the Division of Veterinary Medicine, WRAIR.

### Nucleic acid extraction and next-generation sequencing

The viral cultures for the isolates made in 1968–1970s and stored at -80 °C were used for purification of viral nucleic acids using the QIAamp viral RNA purification kit (Qiagen Sciences, Germantown, MD). The viral culture supernatant was incubated with nucleases to digest free nucleic acids before lysis and extraction of viruses [12]. The purified nucleic acids were used in random reverse transcription using anchored hexamer oligoes followed by random PCR amplification using the anchored hexamer oligoes and primer for the anchor sequence [13]. The PCR amplicons were subjected to next-generation sequencing (NGS) using MiSeq sequencer and reagents including Nextera XT DNA Library Prep kit and MiSeq Reagent kit v3 (Illumina, San Diego, CA).

### Genome sequence assembly and analyses

The sequence data were assembled using Ray de novo genome assembler v2.2 [14] and Roche GS analysis software v2.9 (Roche 454 Life Sciences, Branford, CT, USA). The assembled genome sequences were manipulated and further analyzed using software Geneious version 10.0.9 (Biomatters, Auckland, New Zealand), BLAST programs (http://blast.ncbi.nlm.nih.gov/Blast.cgi), Sequin version 15.50 (https://www.ncbi.nlm.nih.gov/Sequin/) and Molecular Evolutionary Genetics Analysis version 7.0 (MEGA7) [15]. Amino acid sequences were aligned with the MUSCLE program [16] for comparison and phylogenetic analysis. Aligned sequences were used in construction of phylogenetic trees using the maximum-likelihood (ML) method with bootstrap replication of 500 times for calculation of analysis confidence values. The amino acid substitution models for the ML method were compared and the model with the lowest Bayesian Information Criterion (BIC) score was chosen in the analysis [15].

## Results

The recovery and physicochemical characterization of the CaCV viruses and the serological study of the CaCV isolates and the dog cohorts were done during etiological studies of canine diseases in 1963–1978. The whole genome sequencing of the nucleic acids from the archived viral cultures were obtained in 2016.

### Virus recovery and cohort infections

During 1963 through 1978, four similar CPE producing viral isolates, designated as 3–68, L198 T, A128T and W191R, were recovered from two respiratory, a fecal and a penile specimen respectively from military or laboratory dogs. After initial WRCC inoculation, all isolates produced CPE within two to four days. Each of the isolates was re-isolated and readily propagated in these cells. One dog died shortly after collection. The other three dogs developed neutralizing antibodies to their isolates (Table 1). Neutralizing antibody was detected in 20 to 67% of initial serum specimens from each group of dogs and a small percentage of dogs had a rise in titer (Table 1). In addition to these four isolates, three other viruses were recovered from three of these dogs and identified as canine coronavirus co-infected with L198 T, canine picornavirus with A128T [17] and canine parainfluenza virus with W191R, indicating mixed infections occurred in three surviving dogs.

**Table 1:** Antibody neutralization assays for the four canine calicivirus isolates

Rabbit antibody	Neutralization titers				Dog Sera A/P^a^	Serological prevalence	
	3–68	W191R	L198 T	A128T		Arrival^a^	Post^a^
3–68	> 1024	16	< 4	< 4	< 4 / 32	14/21^c^	14/21
W191R	< 16	16,384	< 16	< 16	< 4 / 64	6/30	5/30
L198 T	< 4	< 16	256	4	4 / na^b^	8/29	8/29
A128T	< 4	< 16	< 4	256	< 4 / 16	20/31	21/31^d^

^a^A, arrival titers. P, post titers 14–21 days later

^b^na, not applicable. Post (P) serum not available. Dog died soon after initial specimen collection

^c^The number of sera positive in neutralization assay for each CaCV isolate versus the total number tested

^d^One additional dog showed an increase in titer to A128T

### Chemical and physical characteristics

**Fig. 1:**
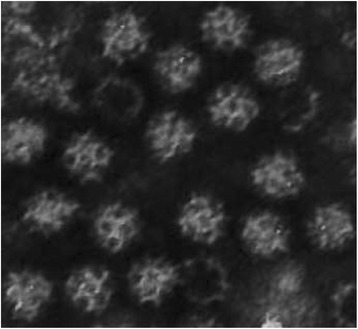
Electron microscopy of negative-stained canine calicivirus isolate W191R. The virus was purified with ultracentrifugation, stained with 2% phosphotungstic acid and examined with a Hitachi HU 12 electron microscope

All four isolates were resistant to chloroform, ether and IUDR treatments, labile at pH 3.0 and were not stabilized at 50 °C in MgCl_2_. The isolates readily passed through membrane filters with a pore size of 50 nm or larger. The buoyant density in cesium chloride for the 3–68 and L198 T isolates were 1.38 and 1.39 respectively. These findings indicate the isolates are non-enveloped RNA viruses with an estimated diameter of 30 nm. Isolates 3–68, L198 T and W191R were partially purified, negative stained and examined with the electron microscope. The viruses were non-enveloped, 42 nm, 36 nm and 30 nm in diameter respectively, which is similar to the 30 nm diameter of picornaviruses (Table 2). However, the isolated viruses have the cup-like surface structure typical of caliciviruses (Fig. 1) [18]. These characteristics are similar to the caliciviruses, e.g., *feline calicivirus* (FCV) [19], vesicular exanthema of swine virus (VESV) [20] and Norwalk-like virus (NLV, *norovirus*) [18].

**Table 2:** Characteristics of canine calicivirus 3–68 and 48 [28]

	3–68 (1968)^a^	48 (1990)^a^
Membrane Filtration	50 μm passage (< 30 nm)	ND^b^
EM	30–42 nm nonenveloped	35–40 nm nonenveloped
Chloroform/ether	nonenveloped	nonenveloped
Density	1.38 g/ml	1.38 g/ml
IUDR	RNA	RNA
pH 3.0	Acid labile	ND
Heat with MgCl2	Heat labile	ND
Hemagglutination assay	Negative at 4 °C, 25 °C, 37 °C, pH 5, pH 7	Negative at 4 °C, 37 °C

^a^Virus name and year of specimen collection. Similar findings to the isolate 3–68 were observed for the other three isolates W191R, L198 T and A128T

^b^ND, not determined

### Antibody neutralization assays

Antibody neutralization assays of the isolates with NIH reference antisera were negative against human poliovirus, echovirus, Coxsackievirus and reoviruses. Assays using antisera to canine viruses including distemper, adenoviruses, herpes, SV5, and canine parvovirus were also negative. The rabbit antisera to each of the isolates were highly specific with 16-fold or higher homologous titers indicating each isolate was antigenically distinct (Table 1). Interestingly, minor non-reciprocal cross reactions occurred between 3 and 68 and W191R and between L198 T and A128T isolates.

### Cell culture susceptibility

All four isolates produced CPE in WRCC and MDCK cell cultures. Attempts to propagate the four isolates in other primary and continuous cell lines were unsuccessful. CPEs were not evident in primary dog kidney or thymus cells or A-72 continuous cells from dog or other animal cells including primary human embryo kidney (HEK), human cell line WI-38, primary cells from African green monkey, rhesus monkey kidney, porcine kidney, feline kidney, rabbit kidney, chicken embryo, hamster kidney and the BHK 21 hamster kidney cell line.

### Genome sequences and sequence comparison with strains 48 and 2117

Nucleic acid extraction was done with 250 μl of the frozen culture of each isolate and used in viral RNA sequencing. Genome sequences for the isolates were de novo assembled and remapped using MiSeq data. The genomes are close to 8.5 kb in length and contain three open-reading frames encoding putative calicivirus non-structural polyproteins (ORF1), major capsid protein VP1 (ORF2) and small capsid protein VP2 (ORF3) (Additional file 1: Figure S1). There are three nucleotides between ORF 1 and ORF 2, which are GCT for strains 48, isolates A128T and L198 T, and GCA for strains 2117, isolates 3–68 and W191R; consequently ORF 1 and 2 are in the same reading frame. ORF2 and ORF3 overlap by four nucleotides ATGA which shifts the reading frame by + 2, in which ATG and TGA are start and stop codon for ORF3 and ORF2, respectively (Additional file 1: Figure S1). The size and organization of ORFs as well as their putative mature proteins are highly consistent with the two representative CaCV strains, strains 48 (NC_004542.1) [21] and 2117 (AY343325) [7] respectively. Nucleotide identity between genome sequences of strain 48 and 2117 is only 71.3%. Genome sequences of strain 48, A128T and L198 T (designated as type I strain) have identities of 87.6–90.7%. Similarly genome sequences of strain 2117, 3–68 and W191R (designated as type II strain) have 86.5–89.0% identity, while the nucleotide sequence identities between these types I and II strains are only 70.3–72.1%. Pairwise multiple amino acid sequence alignments of the conserved RNA-dependent RNA polymerase (RdRP) show high similarity among these viruses, with amino acid identities of 97.5–97.9% within type I, 97.7–98.6% within type II and 71.5–72.4% between types I and II. In contrast, the amino acid identities for the major capsid protein VP1 are 92.0–95.7% in type I, but only 87.1–89.1% in type II and 69.7–71.0% between types I and II (Table 3).

**Table 3:** Comparison of amino acid sequences within and between vesiviruses

A.						
RdRP	FCV	VESV	SMSV	CaCV	CaCV I	CaCV II
FCV	92.5 ± 1.9					
VESV	63.4 ± 1.0	93.4 ± 1.6				
SMSV	59.2 ± 1.0	64.9 ± 1.1	85.5 ± 6.6			
CaCV	57.5 ± 2.5	64.0 ± 2.3	73.6 ± 3.4	85.2 ± 13.3		
CaCV I	54.1 ± 0.9	60.9 ± 0.6	69.0 ± 1.5		97.4 ± 0.5	
CaCV II	59.2 ± 0.7	65.6 ± 0.7	75.9 ± 0.7		72.0 ± 0.7	98.7 ± 0.5
B.						
VP1	FCV	VESV	SMSV	CaCV	CaCV I	CaCV II
FCV	87.4 ± 2.4					
VESV	46.1 ± 0.8	74.2 ± 6.4				
SMSV	38.4 ± 1.2	38.7 ± 1.8	66.4 ± 15.5			
CaCV	37.4 ± 0.8	38.0 ± 1.0	42.3 ± 0.8	78.8 ± 10.5		
CaCV I	36.7 ± 0.5	39.1 ± 0.5	42.6 ± 0.5		92.0 ± 2.9	
CaCV II	37.7 ± 0.8	37.5 ± 0.8	42.2 ± 0.9		67.9 ± 0.9	87.5 ± 3.2

Protein sequences of (A) RNA-dependent RNA polymerase (RdRP) and (B) major capsid protein (VP1) were respectively aligned with MUSCLE program [16]. Data in table are average amino acid identities and standard deviations. GenBank accession numbers of the sequences are shown in Additional file 3: Table S1. FCV, feline calicivirus. VESV, vesicular exanthema. SMSV, San Miguel sea lion virus (SMSV). CaCV, canine calicivirus. CaCV I and II are types I and II of CaCV

### Phylogenetic analysis of vesiviruses

**Fig. 2:**
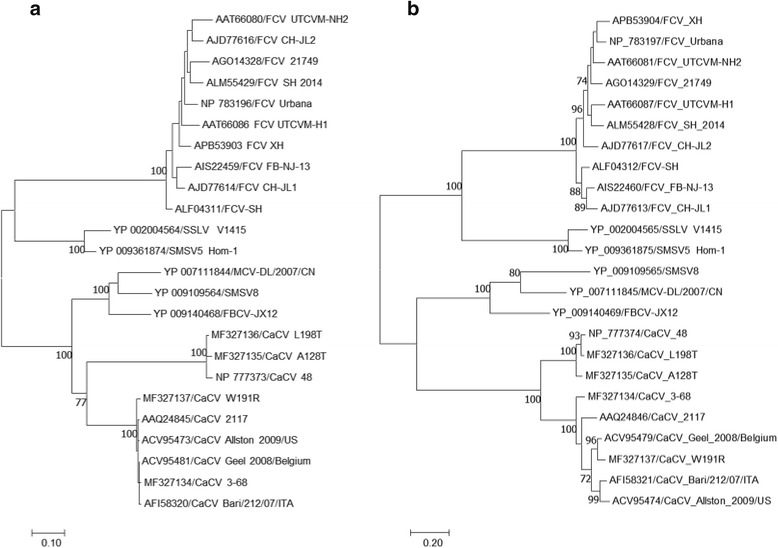
Phylogenetic analysis of canine caliciviruses and related vesiviruses based on complete amino acid sequences of **a** RNA-dependent RNA polymerase (RdRP) and **b** major capsid protein VP1. The selected protein sequences were aligned with the MUSCLE program and used in phylogenetic analyses by the Maximum Likelihood method based on the Le_Gascuel_2008 model. The scale bar represents the number of amino acid substitutions per site. FCV, feline calicivirus. SSLV, Steller sea lion vesivirus. SMSV, San Miguel sea lion virus. MCV, mink calicivirus. FBCV, ferret badger vesivirus

There are two established species in genus *Vesivirus*, FCV and VESV, and additional vesiviruses which are distinct from FCV and VESV and yet to be evaluated. The phylogenetic position of the CaCV isolates were determined based on amino acid sequences of RdRP and VP1 (Fig. 2). Clearly the CaCV isolates A128T and L198 T are close to type I strain 48, while isolates 3–68 and W191R are close to type II strain 2117. These results showed that these CaCVs phylogenetically belong to genus *vesivirus* with clear separation from species FCV and VESV. The known CaCV strains and isolates possibly form two phylogenetic clades. Sequence divergences of CaCV from species FCV or VESV for both RdRP and VP1 proteins are closer to or larger than the distance between FCV and VESV (Table 3). Small genetic variations are seen among viruses within FCV and VESV species, with high amino acid identities of 92.5 ± 1.9% and 93.4 ± 1.6% for RdRP respectively. In contrast, CaCV viruses and San Miguel sea lion viruses (SMSV), despite the small number of identified isolates for each virus, are much more genetically diverse, with RdRP amino acid identities of 85.5 ± 6.6% and 85.2 ± 13.3% respectively. It is interesting that SMSV shared remarkable sequence identities with CaCV, having RdRP amino acid identities of 69.0 ± 1.5% with type I viruses and 75.9 ± 0.7% with type II viruses respectively, comparable to the identity of 72.0 ± 0.7% between types I and II viruses. However, the VP1 sequences differ greatly between SMSV and CaCV, with identity of only 42.2 ± 0.9%, compared to the 65.7% ± 3.4% identity between types I and II viruses. This difference is comparable to the level of difference in VP1 proteins between species FCV and VESV.

### Major capsid protein sequences

ORF2 encodes the major capsid protein precursor of about 690 amino acids. Alignment of amino acid sequences of four CaCV isolates and strains 48 and 2117 (Additional file 2: Figure S2) reveals the existence of conserved motifs, e.g. FRAES (capsid cleavage site), PPG, and the 7-amino-acid CaCV-specific insertion (N/S/K)(S/A/T)IKS(D/S/Q)(I/V) [7, 8] and the existence of multiple potential hyper-variable regions (HVR) at amino acid positions (number for strain 48 VP1) 379−403 (HVR1), 420−458 (HVR2), 467−525 (HVR3), 543−560 (HVR4), and 586−602 (HVR5) (Additional file 2: Figure S2). The antigenically highly distinguishable (Table 1) but genetically highly similar pairs of isolates (Table 3), A128T/L198 T and W191R/3–68 differ by 50 and 89 amino acid residues respectively. Most differences are located in the hyper-variable regions in which the amino acids differ between and within the each pair.

## Discussion

Previous studies of caliciviruses in dogs have reported the recovery of feline calicivirus [22], norovirus [23–25] and sapovirus [26, 27] as well as the candidate canine calicivirus 48 [28] and Bari/212/07/ITA [8]. With the exception of the murine noroviruses, only the vesiviruses can be readily grown in cell culture. This report describes the isolation and identification of four additional canine calicivirus isolates and their molecular characterization. Each of these canine isolates was made solely in the canine WRCC and their canine origin is supported by neutralizing antibody studies in both the dogs providing the isolates and their cohorts (Table 1). The initial physical and chemical studies and electron microscopic observations clearly indicate the isolates are caliciviruses. These characteristics are highly consistent with the few reports since 1985 that establish canine calicivirus as a new virus belonging to the *vesivirus* genus but distinct from FCV and VESV species [28–31].

Neither FCV virus nor antisera for FCV were available to us at the time to examine the serological relationship of these isolates with FCV. Each of the four isolates was antigenically distinct in neutralization tests with only two minor non-reciprocal cross reactions (Table 1).

Determination of each isolate’s genome sequence clearly identified each isolate as a member of the *vesivirus* genus in the *Caliciviridae* family and phylogenetically separate from FCV and VESV species. CaCV apparently has two serologically distinct and genetically divergent types: type I which includes strains 48, isolates A128T and L198 T; and type II which includes strain Bari/212/07/ITA, isolates 3–68 and W191R as well as 2117 and several 2117-like vesiviruses. It is remarkable that CaCV type II viruses share very high amino acid identities for RdRP (98.7% ± 0.5%), but substantially lower identities (87.5 ± 3.2) for VP1. It is rational to speculate that the high divergence of VP1 associates with capsid structure variation, antigenic diversity and in consequence broadened host specificity. It has been shown that vesivirus 2117 has a capsid structure more similar to sapovirus and lagovirus than to vesiviruses and potentially alterations in receptor binding [32]. Vesivirus 2117 [7] and 2117-like viruses Allston 2008/US (GenBank accession GQ475302), Allston 2009/US (GQ475301) and Geel 2008/Belgium (GQ475301) were identified as viral contaminants causative of CPE in production line Chinese hamster ovary (CHO) cells. The origin of these viruses is unknown but possibly from dogs [33]. Vesivirus 2117 can readily grow and develop CPE in MDCK and CHO-K1 cells. CPE in BHK-21 is weak in the first passage and more obvious in the second passage [34]. In a previous study the virus propagated in CHO cells but not in other cells including MDCK and BHK [7]. In our study, isolates 3–68 and W191R were isolated from dogs and maintained in the WRCC dog cell line. Neither produced visible CPE in BHK-21 cultures in first passage. Isolates 3–68 and W191R have respective amino acid identities of 88.7% and 85.7% for VP1 with strain 2117. The divergence of capsid sequences may have contributed to the adaptation of strain 2117 to hamster cells. It is important that the strain 2117-like viruses from CHO cells at three different locations, i.e. Germany, USA and Belgium are significantly different from each other by 14.1 ± 0.9% for VP1 proteins, a similar extent as the distances with other CaCV II viruses. The large heterogeneity of major capsid proteins of CaCV and the evidence of possible cross species infectivity indicates the potential zoonotic transmission of CaCV. Speculation of possible canine to human transmission is supported by the recent finding of Bari/212/07/ITA antibodies in 7.8% of human sera tested in Italy [35].

The serological and molecular assays of CaCV in dogs evidently show a high prevalence of CaCV infection in dogs [33, 36, 37]. In our study, for each isolate we saw rising antibody titers in convalescent sera in comparison with acute sera from each of the three surviving dogs. CaCV antibodies against each isolate were found in acute sera from other dogs in each cohort and only one other dog with rising antibody titer (Table 1). The data clearly suggest the infections by CaCV occurred after the dogs arrived in their cohorts.

Interestingly, the very weak serological cross reactions between the pairs A128T/L198 T and W191R/3–68 can be precisely correlated with the close phylogenetic proximity of each pair. More importantly, the major capsid (VP1) proteins for each pair differ by only a small number of residues, most of which are within HVRs. Further study on these residues may delineate the key antigenic sites responsible for receptor-binding or immunity.

Further genome analyses of the four canine isolates and the previously reported canine 48, Bari/212/07/ITA and Vesivirus 2112 viruses show them forming two clades or species, the first comprising the 48 canine virus with the A128T and L128 T isolates and the second comprising the 2117 and Bari/212/07/ITA canine viruses with the canine 3–68 and W191R isolates (Fig. 2). Examination of the capsid genome of the four isolates and the 48 and 2117 strains reveals HVRs, i.e. nt429 to nt444 which may also be related to the antigenic differences among the newly reported genomes and possibly of significance in the development of specific neutralizing antibodies.

## Conclusions

The physicochemical, serological and molecular study of the four WRAIR canine calicivirus isolates from the late 1960’s and 1970’s have identified the isolates as candidate members of the *Vesivirus* genus of *Caliciviridae*. The detailed analyses demonstrated the restricted cell tropism, antigenic diversity and genetic variation of the CaCV viruses. The genomes of the four isolates together with the previously reported canine 48 and Bari/212/07/ITA strains provide significant additional evidence to support the formation in the canine vesivirus genus of canine calicivirus (CaCV) species consisting of at least two clades/types. Identification of the antigenically distinct vesiviruses capable of growth in cell culture provides a valuable model for studying the role of specific neutralizing antibody in protection against infection and disease during calicivirus infections.

## Additional files

Additional file 1: **Figure S1.** Genome structure of canine calicivirus (CaCV). The CaCV genome is approximately 8.5 kb in length, containing three open-reading frames (*yellow bars*). ORF1 encodes putative non-structural polyprotein precursor which may be cleaved into seven mature proteins (*green bars*), including the RNA-dependent RNA polymerase (RdRP). ORF2 and ORF3 encode major capsid protein (VP1) and small capsid protein (VP2) respectively. There are three nucleotides between the stop codon TGA (in *red*) of ORF1 and the start codon ATG (in *blue*) of ORF2. ORF2 and ORF3 overlap by four nucleotides which contains the start codon (ATG) of ORF3 and stop codon (TGA) of ORF2 respectively. (DOC 61 kb)

Additional file 2: **Figure S2.** Alignment of canine calicivirus (CaCV) major capsid protein VP1 sequences. The MUSCLE program in software Geneious version 10.0.9 was used in multi-sequence alignment of capsid proteins of CaCV type I viruses A128T, L198 T and 48, and type II viruses W191R, 2117, and 3–68. The amino acid residues differing between strains are shown in color. (DOC 559 kb)

Additional file 3: **Table S1.** GenBank accession numbers of the sequences used in Table 3. (XLSX 14 kb)

## Abbreviations

CaCV: Canine caliciviruses
CPE: Cytopathic effects
FBCV: Ferret badger vesivirus
FCV: Feline calicivirus
HVR: Hyper-variable region
ICTV: The International Committee on Taxonomy of Viruses
IUDR: 5-iodo-2-deoxyuridine
MCV: Mink calicivirus
NGS: Next-generation sequencing
PDK: Dog kidney cells
RdRP: RNA-dependent RNA polymerase
SMSV: San Miguel sea lion virus
SSLV: Steller sea lion vesivirus
VESV: Vesicular exanthema of swine virus
WRCC: Walter Reed Canine Cells
