# Supplementary material for: Canine caliciviruses of four serotypes from military and research dogs recovered in 1963−1978 belong to two phylogenetic clades in the Vesivirus genus
Source: Virol J. 2018 Feb 23;15:39. doi: 10.1186/s12985-018-0944-4 (PMC5824495; doi:10.1186/s12985-018-0944-4)
Supplement: Supplementary file 1 — Figure S1. Genome structure of canine calicivirus (CaCV). The CaCV genome is approximately 8.5 kb in length, containing three open-reading frames (yellow bars). ORF1 encodes putative non-structural polyprotein precursor which may be cleaved into seven mature proteins (green bars), including the RNA-dependent RNA polymerase (RdRP). ORF2 and ORF3 encode major capsid protein (VP1) and small capsid protein (VP2) respectively. There are three nucleotides between the stop codon TGA (in red) of ORF1 and the start codon ATG (in blue) of ORF2. ORF2 and ORF3 overlap by four nucleotides which contains the start codon (ATG) of ORF3 and stop codon (TGA) of ORF2 respectively. (DOC 61 kb) [file 12985_2018_944_MOESM1_ESM.doc]

**S1Fig** Genome structure of canine calicivirus (CaCV). The CaCV genome is approximately 8.5 kb in length, containing three open-reading frames (*yellow bars*). ORF1 encodes putative non-structural polyprotein precursor which may be cleaved into seven mature proteins (*green bars*), including the RNA-dependent RNA polymerase (RdRP). ORF2 and ORF3 encode major capsid protein (VP1) and small capsid protein (VP2) respectively. There are three nucleotides between the stop codon TGA (in *red*) of ORF1 and the start codon ATG (in *blue*) of ORF2. ORF2 and ORF3 overlap by four nucleotides which contains the start codon (ATG) of ORF3 and stop codon (TGA) of ORF2 respectively.

**
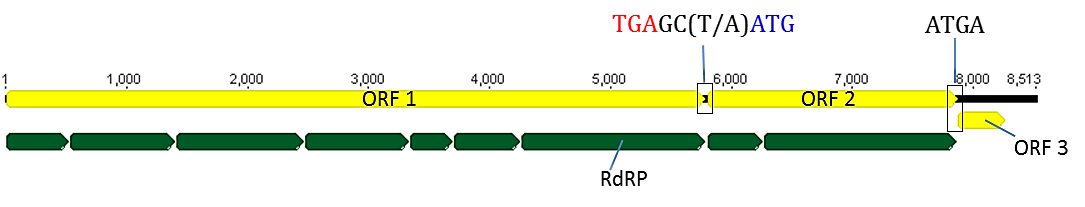
**
