# Supplementary material for: Canine caliciviruses of four serotypes from military and research dogs recovered in 1963−1978 belong to two phylogenetic clades in the Vesivirus genus
Source: Virol J. 2018 Feb 23;15:39. doi: 10.1186/s12985-018-0944-4 (PMC5824495; doi:10.1186/s12985-018-0944-4)

**S2Fig** Alignment of canine calicivirus (CaCV) major capsid protein VP1 sequences. The MUSCLE program in software Geneious version 10.0.9 was used in multi-sequence alignment of capsid proteins of CaCV type I viruses A128T, L198T and 48, and type II viruses W191R, 2117, and 3-68. The amino acid residues differing between viruses are shown in color. The regions in boxes are structural motifs (*blue boxes*) and potential hyper-variable regions (HVR) (*red boxes*).


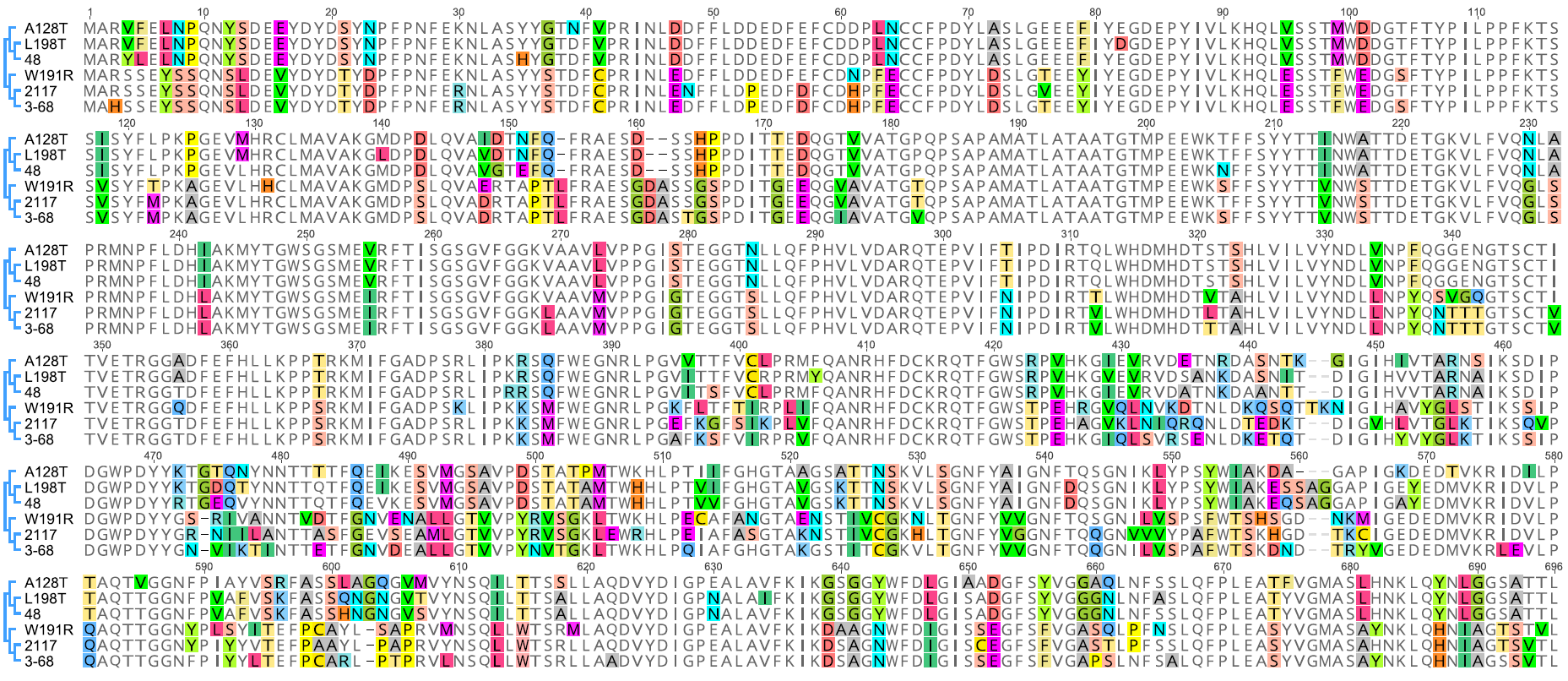

Supplement: Supplementary file 2 — Figure S2. Alignment of canine calicivirus (CaCV) major capsid protein VP1 sequences. The MUSCLE program in software Geneious version 10.0.9 was used in multi-sequence alignment of capsid proteins of CaCV type I viruses A128T, L198 T and 48, and type II viruses W191R, 2117, and 3–68. The amino acid residues differing between strains are shown in color. (DOC 559 kb) [file 12985_2018_944_MOESM2_ESM.doc]
